# Differences in Exercise Performance in Fontan Patients with Extracardiac Conduit and Lateral Tunnel: A FORCE Fontan Registry Study

**DOI:** 10.3390/jcm14124067

**Published:** 2025-06-09

**Authors:** Laura Seese, Mary Schiff, Laura Olivieri, Luciana Da Fonseca Da Silva, Jose P. Da Silva, Adam Christopher, Tyler H. Harris, Victor Morell, Mario Castro Medina, Rahul H. Rathod, Jacqueline Kreutzer, Carlos Diaz Castrillon, Melita Viegas, Tarek Alsaied, the FORCE Investigators

**Affiliations:** Faculty Pavilion, Suite FP5210, UPMC Children’s Hospital of Pittsburgh, 4401 Penn Avenue, Pittsburgh, PA 15224-1334, USA; seeselm@upmc.edu (L.S.); schiffmd@upmc.edu (M.S.); olivierilj@upmc.edu (L.O.); dafonsecadasilval@upmc.edu (L.D.F.D.S.); dasilvajp@upmc.edu (J.P.D.S.); adam.christopher2@chp.edu (A.C.); tyler.harris@chp.edu (T.H.H.); morellvo@upmc.edu (V.M.); castromedinam2@upmc.edu (M.C.M.); rahul.rathod@childrens.harvard.edu (R.H.R.); jacqueline.kreutzer@chp.edu (J.K.); carlos.diazcastrillon@chp.edu (C.D.C.); melita.viegas@cshs.org (M.V.)

**Keywords:** Fontan, exercise testing

## Abstract

**Background:** To explore the differences in exercise capacity between the extracardiac conduit (ECC) and lateral tunnel (LT) Fontan. **Methods:** 2169 patients (36% LT (*n* = 774); 64% ECC (*n* = 1395)) underwent a Fontan operation between 2000 to 2023 in a multi-institutional Fontan registry. LT patients were age-matched to ECC patients, and cardiopulmonary exercise test (CPET) results were compared. Following age-matching and exclusion of those without CPET data, 470 patients emerged with 235 LT and 235 ECC patients. **Results:** ECC achieved higher peak heart rates (174 vs. 169 bpm, *p* = 0.0008) and heart rates at ventilatory anaerobic threshold (VAT) (130 vs. 119 bpm *p* = 0.0005). Oxygen saturations at peak (93.0 vs. 90.0%, *p* = 0.0003) and baseline (95 vs. 92.5%, *p* < 0.0001) were higher in the ECC group. The VO_2_ at VAT was higher in the ECC (17.8 vs. 16.4 mL/kg/min *p* = 0.0123). Baseline pre-exercise heart rate, peak oxygen pulse, VE/VCO_2_ slope, peak VO_2_, peak % of predicted VO_2_, peak work rate, and peak % of predicted work rate were similar (all, *p* > 0.05). Notably, less than 35% of the cohort had a documented CPET. **Conclusions:** We found that the ECC performed statistically better on many parameters of exercise capacity, including the ability to increase heart rate, have higher peak and baseline saturations, and to achieve superior VO_2_ at VAT. However, the magnitude of difference was small, suggesting that the translational value into the clinical realm may be limited. With a minority of the registry patients having CPET completed, this illuminates the need for the implementation of CPET surveillance for Fontan patients.

## 1. Introduction

Since its inception in 1971, the Fontan procedure has drastically enhanced and extended the lives of many patients with single-ventricle congenital heart disease [1,2]. Surgical techniques, perioperative care, and catheter-based interventions have all significantly evolved over the past five decades, allowing many Fontan patients to survive until adulthood [3,4]. With longer follow-up, we have been able to appreciate the challenges that Fontan patients face, including subnormal exercise capacity compared to the biventricular population, a difference that is exacerbated over time [5]. The decrease in exercise capacity in the Fontan population can be attributed to the impaired chronotropic responses and the inability to increase stroke volume in the absence of the subpulmonary ventricle and elevated pulmonary vascular resistance [6,7]. These can be further impaired by diastolic dysfunction, high Fontan pressures, and atrioventricular valve incompetence [8]. Moreover, varying degrees of cyanosis related to fenestration patency, baffle leaks, and veno-venous collateral burden contribute to the inability to exercise at normal levels [9]. As this cycle progresses, these patients are faced with increased rates of deconditioning, reduced skeletal muscle mass, and sarcopenia that further limit exercise tolerance [10].

Cardiopulmonary exercise testing (CPET) is an important prognostication tool for longitudinal Fontan outcomes. As the degree of exercise tolerance (peak VO_2_) decreases to <50% of predicted compared to sex and age-matched healthy peers, Fontan-associated comorbidities become more appreciable, rates of hospitalization increase, and transplant-free survival decreases [11]. Conversely, patients who are high performers in terms of exercise capacity have been shown to have better freedom from comorbidities and enhanced quality of life [12]. The value of CPET testing as a prognosticator is so impactful that the American Heart Association has recommended that Fontan patients undergo serial CPET surveillance [13].

In the modern era, there are two main surgical variations for systemic venous return in the Fontan operation: the lateral tunnel (LT) and the extracardiac conduit (ECC). As the operative mortality following the Fontan operation is now on average < 1.0% in the United States, the impetus to identify modifiable factors that enhance the longitudinal quality of life in Fontan patients has grown [14]. Comparisons of the different types of Fontan circulation have been undertaken, but many of these studies are limited by bias from single-center preferences, inclusion of historical cohorts, and insufficient statistical power. These shortcomings are even more pronounced in studies comparing exercise capacity between the LT and ECC Fontan circulations. Considering the importance of exercise performance on outcomes in the Fontan circulation, we looked to define if a surgically modifiable factor (constructing an LT vs. ECC Fontan circulation) impacted longitudinal exercise performance.

To assess this question, we utilized the Fontan Outcomes Registry using Cardiac Magnetic Resonance (CMR) Examinations (FORCE) registry, which is an international, multicenter consortium of 38 centers that contains over 3900 unique Fontan patients as of 2024. The FORCE registry takes a holistic research perspective, collecting clinical, surgical, diagnostic (CMR, catheterization, echocardiography, CPET, and clinical outcomes data over the patient’s lifetime. The objective of this study was to use the FORCE registry to determine the comparative impact of the LT versus the ECC on exercise capacity in the modern era.

## 2. Methods

### 2.1. Study Design, Patient Population, Data Source, and Definitions

A retrospective study was conducted using data from the multicenter FORCE registry. Approval was obtained by each individual participating institution’s committee on clinical investigations/institutional review board, or via a reliance institutional review board agreement with Boston Children’s Hospital. The study proposal and the manuscript were also approved by the FORCE Data Governance and Publications Committee. The analysis included data received by the FORCE registry as of May 2024. All patients who had a maximal effort CPET (at >8 years of age) recorded in the registry were included in the study. If patients had multiple pairs of exercise testing and CMR examinations, the most recent pair was utilized for analysis. Since the FORCE registry is a CMR database, patients with pre-existing pacemakers are not included in the registry.

### 2.2. Cardiopulmonary Exercise Stress Testing Details

Cardiopulmonary exercise test (CPET) was performed using a calibrated cycle ergometer or treadmill per each institution’s standard protocol. Gas exchange at rest, during exercise, and during recovery was analyzed to determine peak VO_2_. Peak VO_2_ was indexed to weight. Each center in the FORCE registry used its own equation to estimate predicted peak VO_2_. Most commonly, the Wasserman, Cooper et al., and FRIEND (Fitness Registry and the Importance of Exercise: A National Database) equations were used. In our study, all patients had RER ≥ 1.1, indicating maximal effort during cardiopulmonary exercise testing (Table 1) [15,16,17].

### 2.3. Statistical Analysis

**Figure 1:**
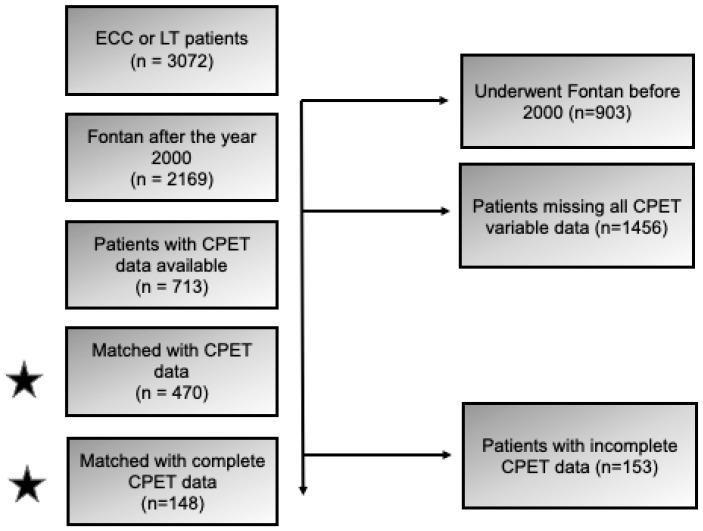
Consort-like diagram depicting study subject inclusion and exclusion criteria.

Data are presented as medians and interquartile ranges (IQR) for continuous variables, and as frequencies and percentages for categorical variables. Many patients did not have all the parameters of the CPET available or did not have CPET completed in the Fontan registry. As such, we identified 713 patients with available CPET data (Figure 1). Characteristics were compared between unmatched ECC and LT groups using Wilcoxon-Mann–Whitney tests for continuous variables, and chi-square or Fisher’s exact tests for categorical variables, as appropriate. ECC patients were then age-matched to LT patients on a 1:1 basis using propensity score matching based on patient age at CPET assessment using a caliper width of 0.2 of the standard deviation of the logit propensity score. We further restricted the sample to only those patients who had complete data on all CPET variables to ensure that the differences observed were not a result of the cohort composition. We then repeated the propensity score matching for this group of LT patients and ECC with complete CPET data for all variables. This resulted in a subgroup of 148 age-matched patients, comprised of 74 LT and 74 ECC. Unless otherwise stated, all tests were two-sided, with an alpha level of 0.05 used to determine statistical significance. SAS version 9.4 (Cary, NC, USA) was used for all analyses.

## 3. Results

There were a total of 3072 patients in the FORCE registry with either ECC or LT at the time of the analysis. There were 903 patients who had their Fontan surgery performed before the year 2000 and were excluded. The CPET data had a high level of missing data overall (~65% per variable). There were 713 total patients who had CPET data available for analysis (Table 2). Additionally, in the unmatched groups, the LT patients were older at the time of CPET (16.2 years vs. 14.8 years, *p* < 0.0001) (Table 3). To ensure the validity of our groups, we age-matched the groups based on the age at CPET (Table 4).

**Figure 2:**
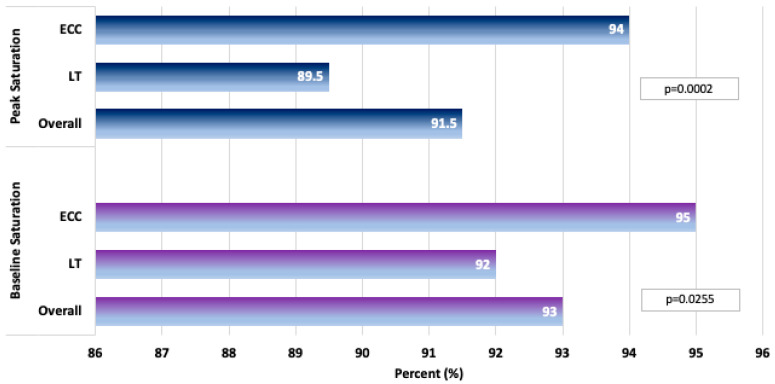
Bar graphs depicting age-matched cardiopulmonary exercise testing outcomes between the lateral tunnel and the extracardiac conduit groups.

From the age-matched CPET groups (235 patients in each group), we found that ECC patients were able to achieve higher peak heart rates (174 vs. 169 bpm, *p* = 0.0008), percent of predicted peak heart rates (86.0% vs. 85.0%, *p* = 0.0354), and heart rate at ventilatory anaerobic threshold (VAT) (130 vs. 119 bpm, *p* = 0.0005) (Table 5; Figure 2). The peak oxygen saturations (93.0% vs. 90.0%, *p* = 0.0003) and the baseline pre-exercise saturations (95.0% vs. 92.5%, *p* < 0.0001) were also higher in the ECC group. Furthermore, the maximal oxygen consumption (VO_2_) at VAT was higher in the ECC group as well (17.8 vs. 16.4 mL/kg/min, *p* = 0.0123). The remaining exercise stress test parameters were statistically similar between the LT and ECC groups. These included baseline pre-exercise heart rate, peak oxygen pulse, peak respiratory exchange ratio (RER), minute ventilation-to-carbon dioxide output (VE/VCO_2_) slope, peak VO_2_, % of peak predicted VO_2_, peak work rate, and % of peak predicted work rate (all, *p* > 0.05). CMR variables were evaluated for this cohort and demonstrated that single ventricle end diastolic volume indexed on body surface area (BSA) (105.6 vs. 96.5 mL/m^2^, *p* = 0.0017) and single ventricle end systolic volume indexed on BSA were higher in the LT group (51.8 vs. 46.8 mL/m^2^, *p* = 0.0065) (Table 6). Importantly, single ventricle ejection fraction and rate of AVV regurgitation were similar between the groups (*p* > 0.05).

**Figure 3:**
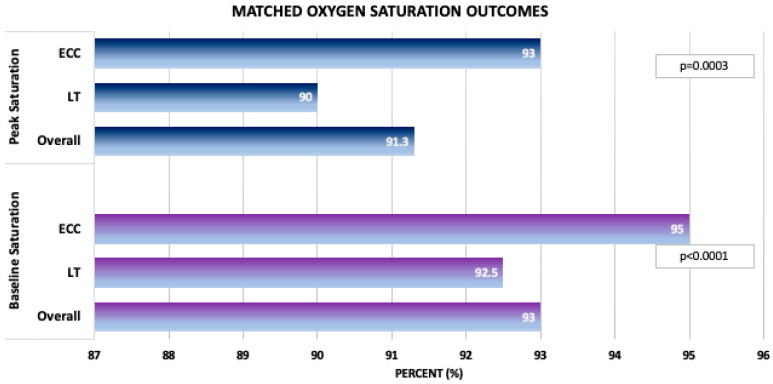
Bar graphs depicting propensity-score-matched oxygen saturations between the lateral tunnel and extracardiac conduit groups with complete data.

**Figure 4:**
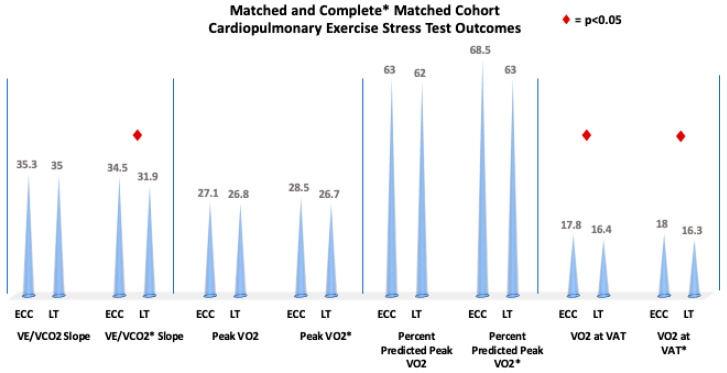
Bar graphs depicting propensity-score-matched cardiopulmonary exercise testing variables between the lateral tunnel and extracardiac conduit groups with incomplete and complete data. Asterisk indicates complete CPET data result.

To ensure that such differences were not solely driven by cohort composition across different outcomes, we evaluated a cohort of age-matched patients with complete CPET data for all parameters (Table 7). This cohort of 148 patients, split into two groups of 74 ECC and 74 LT patients (well matched, with an SMD = 0.0857,), demonstrated similar findings to the larger, original, age-matched groups of CPET patients. The exceptions were that in the complete CPET data groups, the peak heart rate and peak predicted heart rate were statistically higher in the ECC, whereas in the general CPET group, they were similar. The variables of heart rate at VAT (125.5 vs. 116.0 bpm, *p* = 0.0291), peak saturation (94.0% vs. 89.5%, *p* = 0.0002), baseline saturations (95.0% vs. 92.0%, *p* = 0.0255), and VO_2_ at VAT (18.0 vs. 16.3 mL/kg/min, *p* = 0.0044) remained significantly higher in the ECC group compared to the LT group (Figure 3). Furthermore, patients with complete matched data were found to have significantly higher peak work rate (118.5 vs. 112.0 watts, *p* = 0.0443) and percent of predicted work rate (72.5% vs. 66.5%, *p* = 0.0141) in the ECC group compared to the LT group, whereas these parameters were statistically similar in the original matched cohort. ECC patients also had a lower VE/VCO_2_ slope compared to the LT group (31.9 vs. 34.5, *p* = 0.0071) (Figure 4).

## 4. Comment

The Fontan procedure has undergone three major iterations that have resulted in a reduction of major postoperative morbidities and mortality [18,19]. In the modern era, the most widely adopted techniques for total cavopulmonary connection in the Fontan operation are the LT, described in 1988, and the ECC, described in 1990. With these operations evolving over the same time frame, there have been many studies that have attempted to establish the superiority of one approach over another in terms of early comorbidities and mortality [3,19,20,21,22]. The last three decades of evidence have failed to demonstrate a clear superiority of one approach over the other. However, from these studies, we have established the importance of fluid dynamics in reducing substantial energy loss and derived the importance of implanting adequately (>16 mm) sized conduits in the ECC as well as avoiding maldistribution of pulmonary blood flow, and dilation of the lateral tunnel [21]. The negative impacts of arrhythmias, sinus node function, thromboembolic events, and the potential benefits of fenestrations have also been well documented [23,24,25]. In the most recent 2023 Update on Outcomes and Research from the STS congenital cardiac surgery database, the early mortality after the Fontan operation was reported as a median of <1.0% (IQR 0–1.25) nationally [14]. Therefore, with excellent contemporary survival and an increasing number of Fontan patients surviving to adulthood, the research focus has begun to shift towards prognostication for future Fontan failure, transplant-free survival, and quality of life.

Exercise performance is defined by the ability of the body’s cardiopulmonary and muscular unit to utilize oxygen and remove carbon dioxide, and this can be measured by CPET [11]. Fontan patients have a reduced exercise capacity that can be attributed to a lack of a subpulmonary ventricle, chronotropic incompetence, cyanosis, intrapulmonary shunting, lung restriction, reduced skeletal muscle mass, obesity, and deconditioning [26,27]. The results of CPET testing in the Fontan population are valuable in their ability to identify patients at increased risk for morbidity and mortality [11]. Cunningham and colleagues demonstrated that a decline in peak oxygen consumption between consecutive CPETs predicts increased risk for death after transplant in adult Fontan patients, independent of their baseline [28]. These findings were echoed by Ohuchi et al., who showed in a cohort of 335 Fontan patients that those with reduced CPET variables had markedly higher rates of unscheduled hospital admissions and mortality [29]. Furthermore, Egbe and colleagues reported that a decline in percent predicted peak VO_2_ ≥ 3 percentage points/year predicted cardiovascular adverse events in Fontan patients [30]. While these studies herald the importance of serial CPET screening for prognostication and exercise training in Fontan patients, direct comparative studies have yet to establish clear superiority of the LT or the ECC Fontan in terms of exercise performance.

For instance, Bossers and colleagues from the Netherlands evaluated 101 adolescent Fontan patients who were divided into intraatrial LT and ECC cohorts [31]. They found that the LT patients had a lower percentage of predicted peak oxygen uptake and a greater VE/VCO_2_ slope. However, when patients with baffle leaks were excluded, these differences were no longer statistically significant. The group from Boston Children’s Hospital reported on a cohort of 801 patients (*n* = 638 LT and *n* = 163 ECC) and found that both groups performed equally on all parameters except for peak work rate and peak oxygen pulse, which were higher in the LT group [32]. Alsaied et al. evaluated 813 patients who were stratified into high, middle, and low performing groups based on percent predicted VO_2_ [33]. They found that the higher performing groups were inversely associated with mortality and progression to transplantation. However, Fontan type (LT versus ECC) did not contribute to allocation in the higher or lower performing groups. Other studies have also evaluated the impact of age on longitudinal Fontan exercise performance and found that younger age at Fontan completion enhanced exercise performance but did not identify a difference between ECC and LT [34].

In this present study, we compared the exercise performance of 470 age-matched adolescent Fontan patients who were stratified by LT and ECC connection. We found that ECC patients outperformed the LT patients in terms of peak heart rate, peak predicted heart rate, heart rate at VAT, peak saturation, baseline saturation, and VO_2_ at VAT. In a sub-analysis of 148 propensity-matched patients with CPET data, these differences persisted, with the ECC outperforming the LT in terms of several exercise parameters. Notably, the LT did not outperform the ECC in any CPET metric. Despite these findings, the magnitude of absolute difference between the groups in each exercise variable is small (Δ < 5 points in most variables). Furthermore, there was also no difference in peak VO_2_, which is the gold standard for measuring exercise capacity [35].

In essence, we must cautiously consider the intersection of statistical significance and clinical significance before suggesting superiority of one approach over the other. Clearly, the ECC patients were able to increase their heart rates more substantially and had higher oxygen saturations compared to their LT counterparts. Although with a narrow magnitude of difference between and no difference in peak VO_2_, we would suggest that overall exercise capacity is clinically noninferior between the groups. This is an important finding considering the sample size. A glaring observation from this study is that despite a multitude of studies delineating the importance of serial CPET in the Fontan population and the 2019 AHA guidelines for surveillance, over 50% of these adolescent Fontan patients had no CPET testing recorded. The impetus to ensure that Fontan patients receive guideline-directed medical surveillance is immense, and the results of this study illuminate that CPET screening is being underutilized in this population.

## 5. Limitations

This study is subject to the inherent limitations of a large database study with a retrospective design. We also do not have data at this current time on the sizing of the ECC Fontan conduits or the dilation of the LT pathways. Details on the burden of veno-venous collateral were also not captured in this analysis and could contribute to exercise intolerance. Furthermore, while information on fenestration patency is available, we do not have data on the presence or degree of baffle leaks. However, studies have evaluated the impact of fenestrations and baffle leaks on exercise capacity and found that when closed, there are higher saturations, but this is at the expense of lower cardiac index [36,37]. Therefore, substantial clinical improvement in exercise capacity has not been delineated. Moreover, the degree of shunting through a patent fenestration or baffle leak while at peak exercise in Fontan patients is physiologically unique to each individual and can limit the interpretation of the outcomes. While cluster robust standard error estimation was utilized to account for center variations, the reasoning on why certain patients underwent exercise testing and others remained unclear. Moreover, this study may favor patients with a greater baseline functional status, as hospitalized or severely deconditioned Fontan patients are unable to complete CPET. Finally, since the FORCE registry is a CMR database, patients who had early pacemaker placement following their Fontan are not included in the registry.

## 6. Conclusions

With refinement of surgical technique, decision-making, and patient management, Fontan outcomes have surpassed expectations in the modern era, with most patients surviving into adulthood. Even with these outcomes, we strive to understand how our surgical decision-making can enhance the lives of patients longitudinally. In this study, we found that ECC patients were able to increase their heart rates more substantially and had higher saturations than LT patients. However, while ECC patients performed slightly better on most CPET variables, the magnitude of the difference was small. Furthermore, there was no difference in peak VO_2_ between the two strategies. As such, the clinical implications of these differences are likely nominal, suggesting that ECC and LT Fontan types have similar exercise capacity. This study also illuminates the need to prioritize CPET surveillance for Fontan patients, as the majority of patients had not received screening.

## Figures and Tables

**Table 1:** Summary of predicted VO_2_ equations.

Equation	Reference	Population	Output
Wasserman	Wasserman, K.; et al. Principles of Exercise Testing and Interpretation, 5th ed., 2011.	Clinical populations (e.g., cardiopulmonary disease)	Predicted peak VO_2_ based on age, sex, weight, height
Cooper	Cooper, K.H. A means of assessing maximal oxygen intake. *JAMA* **1968**, *203*, 201–204.	General adult population (field-based estimation)	Predicted VO_2_ max from treadmill run time (Cooper test)
FRIEND	Kaminsky, L.A.; et al. Updated Reference Standards for Cardiorespiratory Fitness Measured with Cardiopulmonary Exercise Testing: Data from the FRIEND Registry. *Mayo Clin. Proc.* **2022**, *97*, 285–293.	Healthy adults (normative CPET values)	Normative peak VO_2_ values using CPET in healthy adult populations

**Table 2:** Baseline characteristics of unmatched sample, overall and between Fontan surgery groups.

	Overall (*n* = 713)	Lateral Tunnel (*n* = 241)	Extra Cardiac Conduit (*n* = 472)	*p*-Value
Age at Fontan surgery, years	3.2 (2.4, 4.1)	2.4 (2.1, 3.1)	3.5 (2.8, 4.5)	<0.0001
Female	292 (41.0)	86 (35.7)	206 (43.6)	0.0409
Suspected heterotaxy	83 (11.7)	20 (8.3)	63 (13.4)	0.0448
Dominant ventricular morphology				0.0611
Balanced or mixed	110 (15.7)	34 (14.1)	76 (16.5)	
Left	271 (38.6)	82 (34.0)	189 (41.0)	
Right	321 (45.7)	125 (51.9)	196 (42.5)	
Cardiac diagnosis				<0.0001
Atrioventricular canal defect	51 (7.2)	8 (3.3)	43 (9.1)	
Double inlet left or right ventricle	81 (11.4)	29 (12.0)	52 (11.0)	
Double outlet right ventricle	77 (10.8)	22 (9.1)	55 (11.7)	
Ebsteins	9 (1.3)	4 (1.7)	5 (1.1)	
Hypoplastic left heart syndrome or small left-sided structures	239 (33.5)	107 (44.4)	132 (28.0)	
Mitral atresia (including mitral atresia with DORV)	18 (2.5)	6 (2.5)	12 (2.5)	
Pulmonary atresia with intact ventricular septum	50 (7.0)	9 (3.7)	41 (8.7)	
Tricuspid atresia	116 (16.3)	41 (17.0)	75 (15.9)	
Other hypoplastic right ventricle or small right-sided structures	16 (2.2)	6 (2.5)	10 (2.1)	
Other	56 (7.9)	9 (3.7)	47 (10.0)	
Year of Fontan surgery	2007 (2004, 2010)	2006 (2003, 2009)	2008 (2005, 2011)	<0.0001

Abbreviations: DORV, double outlet right ventricle.

**Table 3:** Unmatched cardiopulmonary CPET characteristics, overall and between Fontan surgery groups.

	Overall	LT	ECC	*p*-Value
Age at time of exercise stress test visit (years)	15.3 (12.7, 18.0) *n* = 713	16.2 (13.6, 19.0) *n* = 241	14.8 (12.3, 17.6) *n* = 472	0.0001
Peak heart rate (beats/minute)	172 (160, 181) *n* = 683	169 (155, 179) *n* = 237	173 (160, 184) *n* = 446	0.0003
Percent of predicted peak heart rate (beats/minute)	86 (80, 90) *n* = 647	85.5 (78, 90) *n* = 228	86 (80, 90) *n* = 419	0.1984
Baseline/Pre-exercise heart rate (beats/minute)	86 (75, 97) *n* = 669	86 (76, 96) *n* = 237	86 (75, 98) *n* = 432	0.8981
Heart rate at VAT (beats/minute)	125.0 (110.0, 143.5) *n* = 408	119.0 (103.0, 133.0) *n* = 163	132.0 (113.0, 148.0) *n* = 245	<0.0001
Peak O_2_ pulse (%)	8.3 (6.7, 11.0) *n* = 455	8.9 (6.6, 11.1) *n* = 175	8.2 (6.8, 10.6) *n* = 280	0.3319
Peak RER	1.16 (1.10, 1.23) *n* = 553	1.15 (1.11, 1.23) *n* = 204	1.16 (1.10, 1.23) *n* = 349	0.6836
Peak saturation (%)	91 (88, 95) *n* = 564	90 (87, 94) *n* = 201	92 (88, 95) *n* = 363	0.0011
Baseline/Pre-exercise saturation (%)	94 (91, 96) *n* = 552	92 (90, 95) *n* = 201	94 (91, 97) *n* = 351	<0.0001
VE/VCO_2_ slope	35.0 (31.0, 39.8) *n* = 503	34.7 (31.0, 40.0) *n* = 179	35.0 (31.0, 39.5) *n* = 324	0.5758
Peak VO_2_ (mL/kg/min)	27.7 (22.2, 33.0) *n* = 638	27.0 (22.0, 31.1) *n* = 223	28.2 (22.4, 34.2) *n* = 415	0.0237
Percent of predicted peak VO_2_ (%)	64 (53, 76) *n* = 570	62 (53, 72) *n* = 199	65 (53, 77) *n* = 371	0.0992
VO_2_ at VAT (mL/kg/min)	17.6 (14.1, 21.9) *n* = 475	16.3 (13.4, 19.3) *n* = 177	19.0 (14.5, 22.6) *n* = 298	<0.0001
Peak work rate (Watts)	106 (85, 141) *n* = 387	112 (84, 145) *n* = 146	104 (85, 137) *n* = 241	0.4825
Percent of predicted work rate (%)	70.0 (59.0, 81.0) *n* = 305	68.0 (55.0, 77.0) *n* = 129	72.0 (60.5, 82.5) *n* = 176	0.0187

Abbreviations: RER, respiratory exchange ratio; VAT, ventilatory anaerobic threshold; VE/VCO_2,_ minute ventilation to carbon dioxide output; VO_2_ maximal oxygen consumption.

**Table 4:** Standardized mean differences (SMD) in age at CPET assessment between matched LT and ECC pairs for the samples in Table 4.

Age at CPET Assessment (Years)	LT	ECC	SMD
Peak heart rate (beats/minute)	16.2 (13.6, 19.0) *n* = 235	16.3 (13.6, 18.7) *n* = 235	0.0274
Percent of predicted peak heart rate sample (%)	16.1 (13.5, 18.9) *n* = 226	16.1 (13.5, 18.5) *n* = 226	0.0301
Baseline/Pre-exercise heart rate (beats/minute)	16.1 (13.5, 18.9) *n* = 234	16.2 (13.5, 18.5) *n* = 234	0.0297
Heart rate at VAT (beats/minute)	16.3 (13.6, 18.9) *n* = 157	16.3 (13.6, 18.3) *n* = 157	0.0724
Peak O2 pulse (mL O_2_/beat)	16.4 (13.8, 19.3) *n* = 170	16.4 (13.8, 18.5) *n* = 170	0.0665
Peak RER sample	16.4 (13.9, 19.3) *n* = 202	16.4 (13.9, 19.1) *n* = 202	0.0274
Peak saturation (%)	15.8 (13.3, 18.8) *n* = 199	15.8 (13.3, 18.8) *n* = 199	0.0177
Baseline/Pre-exercise saturation (%)	16.1 (13.4, 18.8) *n* = 198	15.9 (13.4, 18.8) *n* = 198	0.0199
VE/VCO_2_ slope	16.4 (14.0, 19.4) *n* = 177	16.4 (14.0, 18.8) *n* = 177	0.0461
Peak VO_2_ (mL/kg/min)	16.3 (13.8, 18.9) *n* = 221	16.3 (13.8, 18.8) *n* = 221	0.0199
Percent of predicted peak VO_2_ (%)	16.3 (13.8, 19.3) *n* = 197	16.3 (13.8, 19.3) *n* = 197	0.0155
VO_2_ at VAT (mL/kg/min)	16.4 (13.8, 18.9) *n* = 168	16.4 (13.8, 18.1) *n* = 168	0.0697
Peak work rate (Watt)	15.6 (12.5, 18.0) *n* = 138	15.6 (12.5, 17.9) *n* = 138	0.0454
Percent of predicted work rate (%)	15.3 (12.5, 17.8) *n* = 111	15.4 (12.5, 17.3) *n* = 111	0.0619

Abbreviations: RER, respiratory exchange ratio; VAT, ventilatory anaerobic threshold; VE/VCO_2,_ minute ventilation to carbon dioxide output; VO_2_ maximal oxygen consumption.

**Table 5:** Cardiopulmonary exercise stress test characteristics, overall and between Fontan surgery groups, in unique age-matched samples.

	Overall	LT	ECC	*p*-Value
Peak heart rate (beats/minute)	171 (160, 181) *n* = 470	169 (153, 179) *n* = 235	174 (162, 184) *n* = 235	0.0008
Percent of predicted peak heart rate sample (%)	86 (80, 90) *n* = 452	85 (78, 90) *n* = 226	86 (81, 91) *n* = 226	0.0354
Baseline/pre-exercise heart rate (beats/minute)	86 (75, 97) *n* = 468	86 (76, 96) *n* = 234	86 (75, 97) *n* = 234	0.9036
Heart rate at VAT (beats/minute)	124 (107, 140) *n* = 314	119 (104, 134) *n* = 157	130 (111, 145) *n* = 157	0.0005
Peak O_2_ pulse (mL O_2_/beat)	9.0 (6.8, 11.0) *n* = 340	8.7 (6.5, 11.0) *n* = 170	9.0 (7.0, 11.0) *n* = 170	0.6015
Peak RER sample	1.16 (1.10, 1.23) *n* = 404	1.15 (1.11, 1.22) *n* = 202	1.17 (1.10, 1.23) *n* = 202	0.8397
Peak saturation (%)	91.3 (88.0, 95.0) *n* = 398	90.0 (87.0, 94.0) *n* = 199	93.0 (89.0, 95.0) *n* = 199	0.0003
Baseline/pre-exercise saturation (%)	93.0 (91.0, 96.0) *n* = 396	92.5 (90.0, 95.0) *n* = 198	95.0 (91.0, 97.0) *n* = 198	<0.0001
VE/VCO_2_ slope	35.0 (31.0, 40.0) *n* = 354	35.0 (31.0, 40.0) *n* = 177	35.3 (31.0, 39.8) *n* = 177	0.8438
Peak VO_2_ (mL/kg/min)	27.0 (21.8, 32.1) *n* = 442	26.8 (22.0, 30.8) *n* = 221	27.1 (21.7, 32.8) *n* = 221	0.5823
Percent of predicted peak VO_2_ (%)	62.5 (52.0, 73.0) *n* = 394	62.0 (53.0, 72.0) *n* = 197	63.0 (52.0, 74.0) *n* = 197	0.9619
VO_2_ at VAT (mL/kg/min)	16.8 (13.4, 20.6) *n* = 336	16.4 (13.4, 19.5) *n* = 168	17.8 (13.4, 22.0) *n* = 168	0.0123
Peak work rate (Watt)	112.0 (85.0, 144.0) *n* = 276	112.0 (83.0, 143.0) *n* = 138	112.5 (87.0, 144.0) *n* = 138	0.5080
Percent of predicted work rate (%)	69.5 (59.0, 82.0) *n* = 222	68.0 (56.0, 79.0) *n* = 111	71.0 (60.0, 83.0) *n* = 111	0.1325

Abbreviations: RER, respiratory exchange ratio; VAT, ventilatory anaerobic threshold; VE/VCO_2,_ minute ventilation to carbon dioxide output; VO_2_ maximal oxygen consumption.

**Table 6:** Cardiac magnetic resonance imaging variables between Fontan surgery groups, in unique age-matched samples.

	LT *n* = 235	ECC *n* = 235	*p*-Value
Body mass index	20.4 (18.5, 23.6)	20.5 (17.8, 24.0)	0.3730
Ascending aorta, above the Stansel flow rate, indexed to BSA	2.9 (2.5, 3.4)	3.0 (2.5, 3.5)	0.1678
Fontan flow indexed on BSA	1.6 (1.2, 2.0)	1.5 (1.2, 1.9)	0.0521
Qp/Qs	1.0 (0.9, 1.2)	1.0 (0.9, 1.2)	0.5423
Aortic or native aortic regurgitation fraction	4.0 (2.0, 6.0)	4.0 (2.0, 8.0)	0.9568
Mitral or common atrioventricular valve regurgitation fraction	10.0 (6.0, 15.0)	15.0 (5.0, 23.0)	0.0983
Pulmonary or neo-aortic regurgitation fraction	5.0 (3.0, 6.0)	5.0 (2.0, 11.0)	0.4670
Tricuspid regurgitation fraction	17.0 (12.0, 25.0)	14.0 (7.0, 27.0)	0.1942
Single ventricle end diastolic volume indexed on BSA	105.6 (85.9, 128.8)	96.5 (78.7, 121.0)	0.0017
Single ventricle ejection fraction	51.2 (44.9, 55.9)	51.7 (45.4, 57.5)	0.1739
Single ventricle end systolic volume indexed on BSA	51.8 (39.0, 66.5)	46.8 (34.9, 62.7)	0.0065
Single ventricle mass indexed on BSA	52.6 (41.2, 66.2)	50.6 (38.6, 67.2)	0.2554
Visible aortopulmonary collaterals	47 (25.7)	79 (20.4)	0.1569
Visible veno-venous collaterals	27 (15.3)	57 (14.0)	0.7009

Abbreviations: BSA, body surface area.

**Table 7:** Cardiopulmonary exercise testing, overall and between Fontan surgery groups, in propensity-score-matched sample with non-missing data on all exercise testing parameters and matching variables.

	Overall (n = 148)	LT (n = 74)	ECC (n = 74)	*p*-Value
Peak heart rate (beats/minute)	166 (157, 179)	166 (153, 178)	171 (160, 181)	0.1221
Percent of predicted peak heart rate sample (%)	86.0 (79.5, 91.0)	85.5 (76.0, 90.0)	87.0 (81.0, 92.0)	0.1939
Baseline/pre-exercise heart rate (beats/minute)	87 (78, 97)	89 (79, 97)	86.5 (78, 98)	0.7386
Heart rate at VAT (beats/minute)	118.5 (105.0, 136.0)	116.0 (103.0, 131.0)	125.5 (111.0, 140.0)	0.0291
Peak O_2_ pulse (mL O_2_/beat)	8.2 (7.0, 11.0)	8.0 (6.6, 10.1)	9.0 (7.0, 11.0)	0.0769
Peak RER sample	1.19 (1.12, 1.27)	1.14 (1.11, 1.22)	1.23 (1.15, 1.30)	0.0032
Peak saturation (%)	91.5 (88.0, 94.5)	89.5 (86.0, 93.0)	94.0 (90.0, 96.0)	0.0002
Baseline/pre-exercise saturation (%)	93 (90, 97)	92 (90, 95)	95 (91, 97)	0.0255
VE/VCO2 slope	33.0 (29.2, 37.4)	34.5 (31.0, 40.0)	31.9 (28.8, 35.3)	0.0071
Peak VO_2_ (mL/kg/min)	27.4 (22.5, 32.7)	26.7 (22.1, 31.3)	28.5 (23.8, 34.5)	0.1208
Percent of predicted peak VO_2_ (%)	65.0 (57.0, 77.0)	63.0 (55.0, 75.0)	68.5 (59.0, 79.0)	0.1300
VO_2_ at VAT (mL/kg/min)	16.7 (13.8, 20.7)	16.3 (13.4, 18.7)	18.0 (14.7, 22.4)	0.0044
Peak work rate (Watt)	113.5 (86.5, 143.5)	112.0 (83.0, 139.0)	118.5 (88.0, 149.0)	0.0443
Percent of predicted work rate (%)	70.0 (59.5, 83.0)	66.5 (56.0, 77.0)	72.5 (63.0, 87.0)	0.0141

Abbreviations: RER, respiratory exchange ratio; VAT, ventilatory anaerobic threshold; VE/VCO_2,_ minute ventilation to carbon dioxide output; VO_2_ maximal oxygen consumption.

## Data Availability

The data presented in this study are openly available in the FORCE Fontan Registry at https://www.forceregistry.org/.

## Supplementary Materials

The following supporting information can be downloaded at: https://www.mdpi.com/article/10.3390/jcm14124067/s1. Table S1: List of Fontan Outcomes Registry using Cardiac Magnetic Resonance (CMR) Examinations (FORCE) registry investigators.
