# Supplementary material for: Differences in Exercise Performance in Fontan Patients with Extracardiac Conduit and Lateral Tunnel: A FORCE Fontan Registry Study
Source: J Clin Med. 2025 Jun 9;14(12):4067. doi: 10.3390/jcm14124067 (PMC12193827; doi:10.3390/jcm14124067)
Supplement: Supplementary file 1 [file jcm-14-04067-s001.zip › jcm-3642788-supplementary.pdf]

Supplemental Table S1. List of **F**ontan **O**utcomes **R**egistry using **C**ardiac Magnetic **R**esonance (CMR) **E**xaminations (FORCE) registry investigators.

**FORCE Investigators**	
**Co-Author**	**Hospital**
Aggarwal, M	Department of Pediatrics, St. Louis Children's Hospital, St. Louis, MO
Alsaied, T	The Heart and Vascular Institute, UPMC Children's Hospital of Pittsburgh, Pittsburgh, PA
Chegond, M	Division of Pediatrics, Stead Family Children's Hospital, Stadfast, IA
Dorfman, AL	Congenital Heart Center, CS Mott Children's Hospital, Ann Arbor, MI
Files, MD	Division of Pediatric Cardiology, Seattle Children's Hopsital, Seattle, WA
Fogel, M	Division of Cardiology, The Children's Hospital of Philadelphia, Pennsylvania, PA
Hegde, S	Division of Pediatric Cardiology, Rady Children's Hospital, San Diego, CA
Krishnamurthy, R	Department of Radiology, Nationwide Children's Hospital, Columbus, OH
Kutty, S	Department of Pediatric and Congenital Cardiology, John Hopkins Hospital, Baltamore, MD
Lam, CZ	Department of Diagnostic and Interventional Radiology, The Hospital for Sick Children, Toronto, Canada
Loke, Y	Division of Cardiology, Children's National Hospital, Washington DC
Marsden, AL	Department of Bioengineering & Pediatrics, Stanford University, Palo Alto, CA
Muthurangu, V	UCL Centre for Cardiovascular Imaging, Institute of Cardiovascular Science, London, UK
Olivieri, LJ	The Heart and Vascular Institute, UPMC Children's Hospital of Pittsburgh, Pittsburgh, PA
Quail, M	UCL Centre for Cardiovascular Imaging, Institute of Cardiovascular Science, London, UK
Raimondi, F	Congenital Cardiology Unit, Ospedale Papa Giovanni XXIII, Bergamo, Italy
Rathod, RH	Department of Cardiology, Boston Children's Hospital, Boston, MA
Renella, P	Division of Pediatric Cardiology, CHOC Children's Hospital, Orange, CA
Renno , MS	Division of Pediatric Cardiology, Arkansas Children's Hospital, Little Rock, AR
Robinson, JD	Division of Pediatric Cardiology, Ann & Robert H. Lurie Children's Hospital, Chicago, IL
Ruchira, G	Division of Pediatric Cardiology, Cedars-Sinai Guerin Children's Hospital, Los Angeles, CA
Shah, A	Division of Pediatric Cardiology, New York-Presbyterian Morgan Stanley Children's Hospital, New York, NY
Slesnick, TC	Division of Pediatric Cardiology, Children's Healthcare of Atlanta, Atlanta, GA
Soslow, JH	Division of Pediatric Cardiology, Vanderbilt University Medical Center, Nashville, TN
Steele, J	Division of Pediatric Cardiology, Yale New Haven Children's Hospital, New Haven, CT
Stern, KW	Division of Pediatric Cardiology, Mount Sinai Kravis Children's Hospital, New York, NY
Tiffanie, B	Division of Pediatric Cardiology, Riley Hospital for Children, Indianapolis, IN

Vaikom House, A	Division of Pediatric Cardiology, Oklahoma Children's Hospital, Oklahoma City, OK
Weigand, J	Division of Pediatric Cardiology, Texas Children's Hospital, Houston, TX
